# The Desaturase Gene *Nlug-desatA2* Regulates the Performance of the Brown Planthopper *Nilaparvata lugens* and Its Relationship with Rice

**DOI:** 10.3390/ijms21114143

**Published:** 2020-06-10

**Authors:** Wenfeng Ye, Jiamei Zeng, Wenhui Hu, Carlos Bustos-Segura, Ali Noman, Yonggen Lou

**Affiliations:** 1 State Key Laboratory of Rice Biology, Institute of Insect Science, Zhejiang University, Hangzhou 310058, China; wenfeng.ye@unine.ch (W.Y.); tsengchiamei@zju.edu.cn (J.Z.); hwhsntu@outlook.com (W.H.); alinoman@gcuf.edu.pk (A.N.); 2 Laboratory of Fundamental and Applied Research in Chemical Ecology, University of Neuchâtel, 2000 Neuchâtel, Switzerland; carlos.bustos@unine.ch; 3 Department of Botany, Government College University, Faisalabad 38040, Pakistan

**Keywords:** *Nilaparvata lugens*, desaturase, fecundity, rice, herbivore-induced defense responses

## Abstract

Insect desaturases are known to play an important role in chemical communication between individuals. However, their roles in insect growth, development and fecundity, and in regulating interactions of insects with plants, remain largely unknown. In this study, we explored the functions of *Nlug-desatA2*, a desaturase gene of the brown planthopper (BPH), *Nilaparvata lugens* (Stål). The RNA interference-based knockdown of *Nlug-desatA2* decreased the ratio of monounsaturated fatty acids to saturated fatty acids, and the level of fatty acids and triglycerides in BPH. *Nlug-desatA2*-knockdown also reduced the food intake, body mass and fecundity of female BPH adults, and led to abdomen atrophy and ovarian agenesis. *Nlug-desatA2*-knockdown suppressed the transcription of *TOR* (target of rapamycin), *Lpp* (Lipophorin) and *AKHR* (adipokinetic hormone receptor) in female adults. Moreover, the corrected survival rate of BPH with *Nlug-desatA2*-knockdown fed an artificial diet was higher than the survival rate of those fed on rice plants. Higher levels of salicylic acid in rice infested by *Nlug-desatA2*-knockdown female BPH adults than in rice infested by control BPH may be the reason. These findings demonstrate that *Nlug-desatA2* has an essential role in lipid metabolism and is involved in the food intake, survival, development and fecundity of BPH. In addition, this gene is likely involved in regulating the responses of rice to BPH infestation.

## 1. Introduction

Desaturases, which catalyze the formation of unsaturated bonds at specific positions in fatty acyl substrates, are essential for a variety of biological processes, including lipid metabolism, the regulation of membrane fluidity and cell signaling [1]. Therefore, they play an important role in the growth, development and fecundity of organisms [2,3]. In insects, desaturases belong to the membrane-bound acyl-coenzyme A (CoA) desaturase category [1]. Insect desaturases are known to facilitate chemical communication between individuals [4,5,6]. Some desaturase genes, for example, have unique functions in the production and perception of sex pheromones [7,8]. The knockdown of the two metathoracic scent gland desaturase genes *Asutdes1* and *Asutdes2* in *Adelphocoris suturalis*, an insect pest that attacks cotton, for example, enhance the production of (E)-4-oxo-2-hexenal, a component of the sex pheromone blend of the insect, and dramatically suppressed the sexual attractiveness of *A. suturalis* females to males [9]. Manipulated transcript levels of *desat1* affect the ability of *Drosophila melanogaster* to both emit and detect pheromones [10]. In addition, some studies have suggested that desaturases influence food intake, development and reproduction in insects. In *D. melanogaster*, desat1 is responsible for inducing the double bond in C16:0 and C18:0, a bond that is needed to produce C16:1 and C18:1 [11]. The deletion or overexpression of *desat1* in *D. melanogaster* larvae, for instance, leads to lipid imbalance and developmental disorder, and can result in a lethal phenotype [12]. The inhibition of desaturases caused by using CAY10566 blocks both feeding intake and the development of *D. melanogaster* [13]. Correspondingly, the developmental lethality of *D. melanogaster* larvae with the knockdown of *desat1* can be rescued by supplementing the diet with fatty acids, especially oleic acid (C18:1) [14]. In *Anopheles coluzzii*, the knockdown of stearoyl CoA desaturase (SCD1) leads to undeveloped ovaries [15]. Further investigation of desaturases is needed to determine their role in the development and fecundity of insects.

Vitellogenesis plays a central role in insect fecundity, including egg development and maturation [16]. During vitellogenesis, vitellogenin (Vg), a critical precursor of the egg yolk protein vitellin (Vn) in many oviparous insects [17,18], is synthesized in the female fat body, secreted into the hemolymph and then incorporated into the developing oocytes via endocytosis mediated by the Vg receptor (VgR) [19,20]. In many insects, vitellogenesis and egg maturation are regulated by the juvenile hormone (JH) pathway [21,22,23] and the nutrition-related target of rapamycin (TOR) pathway [24,25,26]. In the JH pathway, genes for JH biosynthesis (juvenile hormone acid methyltransferase, *JHAMT*) [26,27,28], degradation (juvenile hormone esterase, *JHE*) [29,30] and reception (methoprene-tolerant, *Met*) [31,32] are involved in the regulation of *Vg* transcription as well as ovarian development. In the TOR pathway, both the upstream activator small guanosine triphosphatase (GTPase) Rheb (Ras homologue enriched in brains) [25] and the major downstream target S6K (S6 protein kinase) [33,34] are required for the transduction of nutrition signals and for egg development. The single gene knockdown of *Rheb*, *TOR* or *S6K* curtailed the expression of *Vg* and led to reduced fecundity [25,26,33]. Moreover, some genes involved in lipid transport and nutrition metabolism are thought to influence insect fecundity. Lipophorin (Lpp) is the primary lipid carrier protein in the insect hemolymph. Lipophorin uptake plays a significant role in ovary development and lipid storage in insect oocytes [35,36]. AKH (adipokinetic hormone) and AKHR (adipokinetic hormone receptor) in the AKH pathway cooperate in the regulation of glycogen degradation. AKH regulates the storage of disaccharide trehalose in *D. melanogaster* [37] and indirectly affects egg formation in the two-spotted cricket *Gryllus bimaculatus* [38]. All these signaling pathways and genes, which are interrelated, work together in regulating vitellogenesis and ovary development [16,22,26].

When attacked by herbivorous insects, plants recognize elicitors from herbivorous insects and then activate a defense-related signaling network consisting mainly of jasmonic acid (JA)-, salicylic acid (SA)- and ethylene (ET)-mediated pathways [39,40,41]. The activation of this signaling network induces the production of defensive compounds and hence enhances the resistance of plants to herbivores [42,43]. On the other hand, herbivorous insects adapted to host plants can secrete effectors to inhibit defense responses in plants [44,45,46,47]. During such an interaction, elicitors and effectors derived from herbivorous insects play a vital role. Evidence suggests that the level or activity of elicitors and effectors from an insect can be influenced by many factors, such as its developmental stage [48], diet [49,50], symbionts and the extent to which it is parasitized [51,52,53]. However, whether desaturases affect insect-induced defense responses in plants by influencing the level or activity of insect elicitors and effectors remains unknown.

The brown planthopper (BPH), *Nilaparvata lugens* (Stål) (Hemiptera: Delphacidae), is one of the most destructive pests of rice (*Oryza sativa* L.) in Asia. Recently, we identified ten *Nlug-desat* genes encoding putative desaturases in the BPH genome and demonstrated their essential role in the insect’s survival and fatty acid metabolism [54]. Of these genes, the mean transcript level of *Nlug-desatA2* is higher in the egg stage and in 2- to 4-day-old female adults than in the nymph stage, and its expression peaks in the ovary [54]. Nlug-desatA2, homologous to *D. melanogaster* desat1, possesses a Δ9-fatty acid desaturase-like conserved domain (Δ9-FADS-like, cd03505) and belongs to the first desaturase family [54]. Keeping in mind the significance of desaturases in lipid metabolism and the expression pattern of *Nlug-desatA2*, we, using molecular biology, chemical analysis and bioassays, investigated the role of *Nlug-desatA2* in lipid metabolism, food intake, development and fecundity of BPH, and in regulating the interaction between BPH and rice plants.

## 2. Results

### 2.1. Knockdown of Nlug-desatA2 Changes the Profile of Fatty Acids in BPH Female Adults

**Figure 1:**
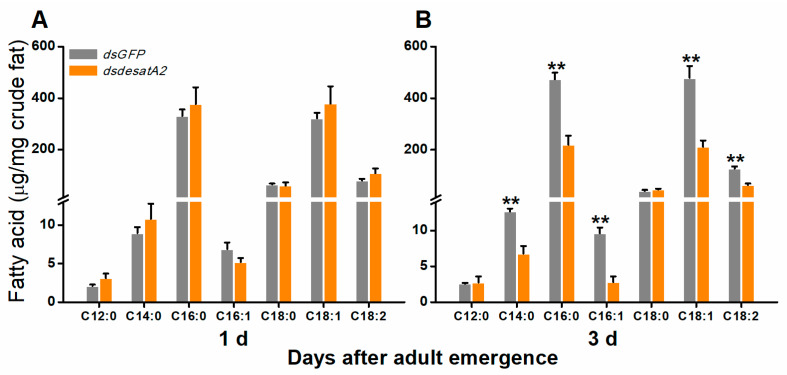
The effect of knocking down *Nlug-desatA2* on fatty acid levels in female BPH adults. Mean levels (+ SE, *n* = 3) of fatty acids per milligram of crude fat from the whole bodies of 1- (**A**) and 3-day-old (**B**) female adults that had been injected with 0.25 µg dsRNA of *Nlug-desatA2* (*dsdesatA2*) or *GFP* (*dsGFP*) at the third-instar nymph stage: C12:0 (lauric acid), C14:0 (myristic acid), C16:0 (palmitic acid), C16:1 (palmitoleic acid), C18:0 (steraic acid), C18:1 (oleic acid) and C18:2 (linoleic acid). Asterisks indicate significant difference between *dsGFP* and *dsdesatA2* injection. (** *p* < 0.01, Student’s *t*-test).

**Figure 2:**
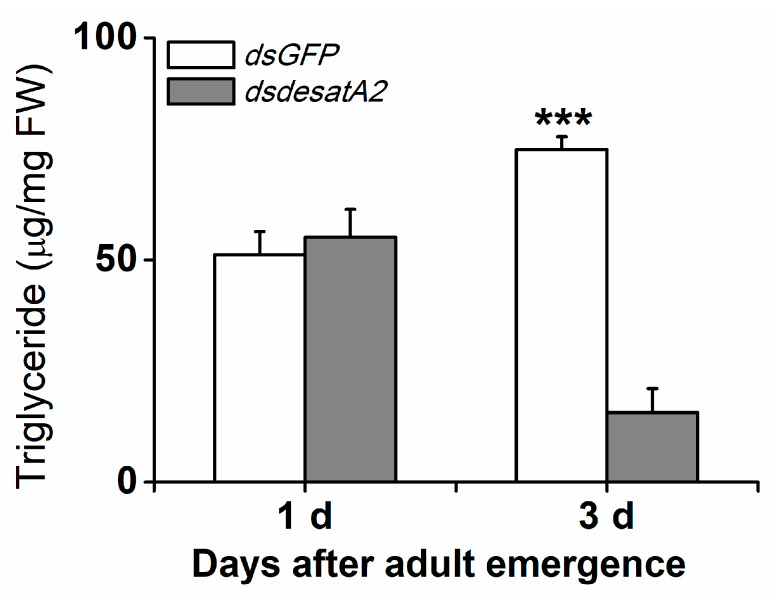
Knocking down *Nlug-desatA2* reduced the amount of triglycerides found in female BPH adults. Mean levels (+ SE, *n* = 3) of triglycerides in whole bodies of 1- or 3-day-old female adults that had been injected with 0.25 µg dsRNA of *Nlug-desatA2* (*dsdesatA2*) or *GFP* (*dsGFP*) at the third-instar nymph stage. Asterisks indicate significant difference between treatments (*** *p* < 0.001, Student’s *t*-test).

To investigate the involvement of *Nlug-desatA2* in the regulation of BPH fatty acid metabolism, we knocked down *Nlug-desatA2* using RNA interference (RNAi), as described in Reference [55], and analyzed the fatty acids in the whole bodies of female BPH adults. Injecting BPH with the 287 bp fragment of *Nlug-desatA2* double-stranded RNA (dsRNA) decreased the transcript levels of *Nlug-desatA2* in the whole body of the insect by 95.20% and 99.84%, 1- and 3-days post-adult emergence, respectively (Figure S1). Seven fatty acids, including four saturated fatty acids (SFAs)—lauric acid (C12:0), myristic acid (C14:0), palmitic acid (C16:0) and stearic acid (C18:0), two monounsaturated fatty acids (MUFAs)—palmitoleic acid (C16:1) and oleic acid (C18:1), and one polyunsaturated fatty acid (PUFA)—linoleic acid (C18:2), were detected in the bodies of BPH female adults (Figure 1). Neither the individual nor the level of fatty acids was influenced in newly emerged dsdesatA2-BPH (1-day-old females) (Figure 1A and Figure S2A). The knockdown of *Nlug-desatA2* reduced the level of individual fatty acids (except for C12:0 and C18:0) and total fatty acids in 3-day-old females. The abundance of total fatty acids in *Nlug-desatA2*-knockdown individuals, compared to that in control individuals, dropped by 47.84% in SFAs and 54.82% in UFAs among 3-day-old female adults (Figure 1B and Figure S2B). The ratio of C16:1 to C16:0 in 1- and 3-day-old dsdesatA2-BPH, and of C18:1 to C18:0 and MUFAs to SFAs in 3-day-old dsdesatA2-BPH, compared with these ratios in dsGFP-BPH, was significantly reduced by 30.98%, 38.92%, 61.44% and 14.89%, respectively; however, the ratio of C18:1 to C18:0 was slightly higher in 1-day-old dsdesatA2-BPH than in dsGFP-BPH (Figure S3). Moreover, *Nlug-desatA2* knockdown reduced the level of triglycerides (TAGs) by 79.06% in 3-day-old females but not in 1-day-old females (Figure 2).

### 2.2. Knockdown of Nlug-desatA2 Impairs BPH Feeding, Growth and Survival

**Figure 3:**
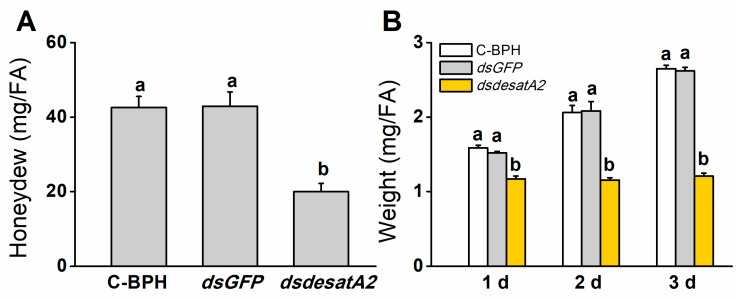
Knocking down *Nlug-desatA2* reduces the feeding and body mass of female BPH adults (FA). (**A**) Mean amount (+ SE, *n* = 19) of honeydew secreted by a newly emerged female BPH adult that had been injected with 0.25 µg dsRNA of *Nlug-desatA2* (*dsdesatA2*) or *GFP* (*dsGFP*), or not injected (C-BPH), at the third-instar nymph stage. The honeydew was weighed 24 h after infestation. (**B**) Mean body mass (+ SE, *n* = 6) of 1- to 3-day-old female adults that received the same treatment as in (**A**). Letters indicate significant differences among different treatments (*p* ˂ 0.05, Duncan’s multiple range test).

**Figure 4:**
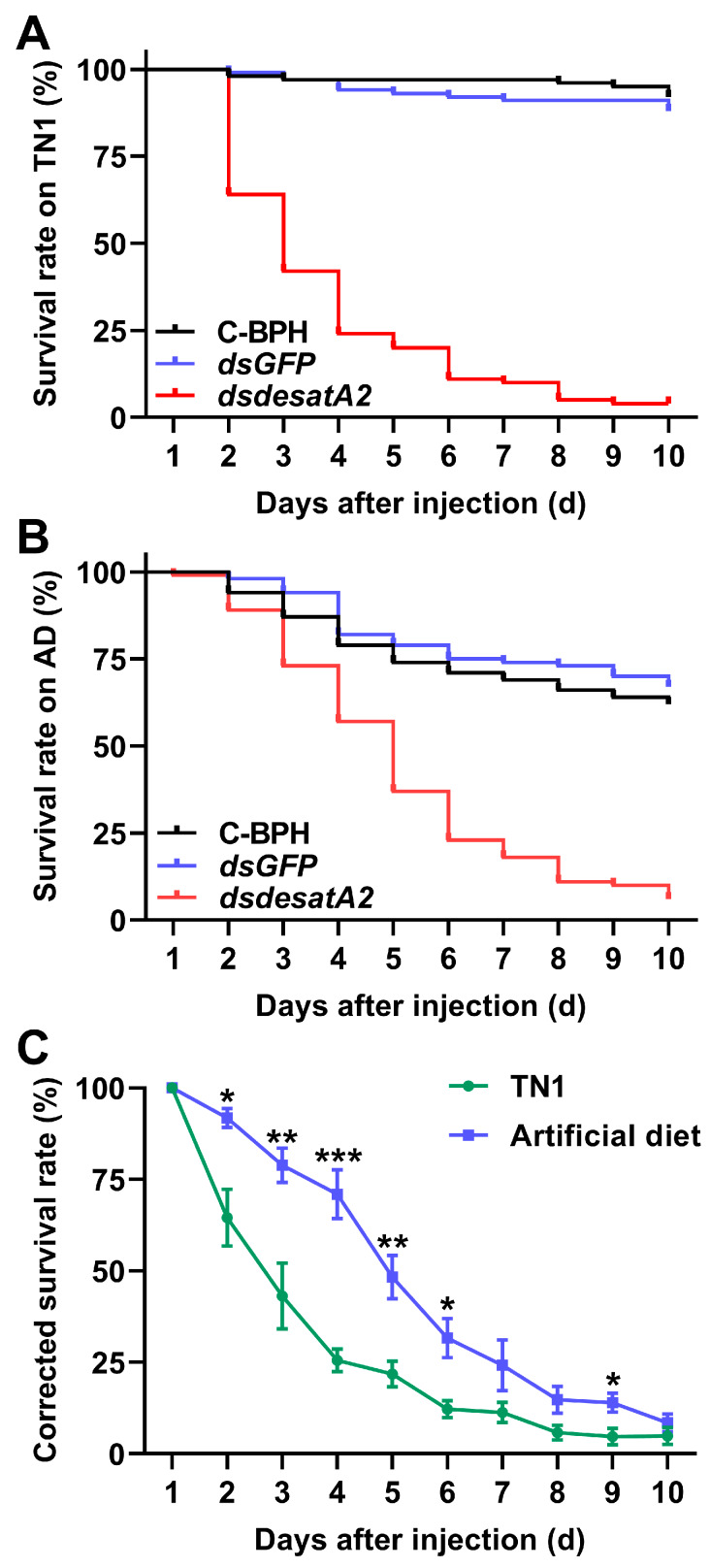
Knocking down *Nlug-desatA2* decreases survival rates of BPH. Survival of BPH (*n* = 20) which were injected with dsRNA of *Nlug-desatA2* (*dsdesatA2*) or *GFP* (*dsGFP*), or not injected (C-BPH), at the third-instar nymph stage and fed rice variety TN1 (**A**) or an artificial diet (**B**). Experiments were performed in five replicates. The statistical significances among different treatments were analyzed with a log-rank (Mantel-Cox) test. (**C**) Mean corrected survival rates (+ SE, *n* = 5) of BPH nymphs injected with *Nlug-desatA2* dsRNA, using BPH nymphs with injected *GFP* dsRNA as controls, fed on rice or an artificial diet. Asterisks indicate significant differences between *dsGFP* and *dsdesatA2* injection. (* *p* < 0.05, ** *p* < 0.01 and *** *p* < 0.001, Student’s *t*-test).

We recorded significantly decreased amounts of honeydew as an index of food intake in dsdesatA2-BPH adults: the amount of honeydew secreted by *Nlug-desatA2*-knockdown individuals, compared to that secreted by C-BPH and dsGFP-BPH, dropped by 53.16% and 53.51%, respectively (Figure 3A). The body masses of dsdesatA2-BPH adults from 1 to 3 days post-adult emergence were comparatively lower than those of C-BPH and dsGFP-BPH adults (Figure 3B). Moreover, the knockdown of *Nlug-desatA2* significantly reduced the survival of BPH on TN1 or artificial diet (*p* < 0.0001, Figure 4A,B). Interestingly, in comparison with the survival rate of *Nlug-desatA2*-knockdown BPH nymphs reared on an artificial diet, the corrected survival rate of BPH nymphs with knockdown *Nlug-desatA2* raised on rice was significantly lower 2 to 6 days post-injection (Figure 4C).

### 2.3. Knockdown of Nlug-desatA2 Blocks Ovary Development in BPH Female Adults

**Figure 5:**
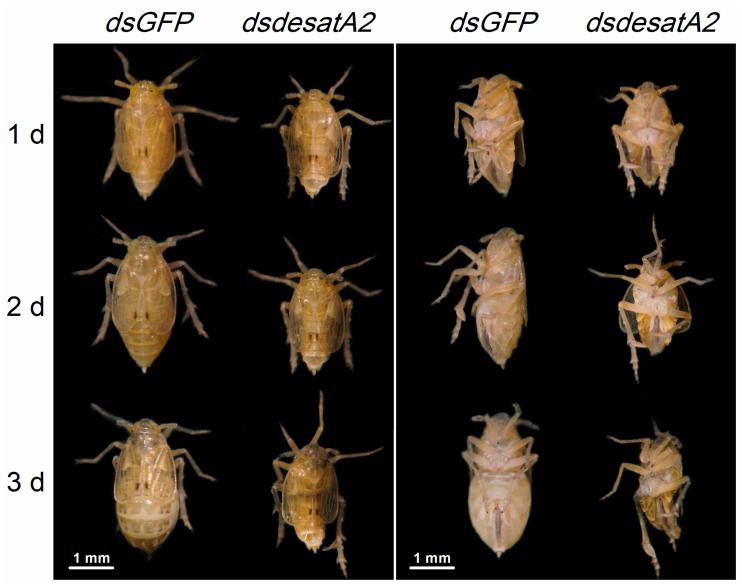
The phenotypes of female BPH adults produced by injection with dsRNA for *Nlug-desatA2*. Third-instar nymphs were injected with 0.25 µg dsRNA of *GFP* (*dsGFP*) or *Nlug-desatA2* (*dsdesatA2*). Female BPH adults were collected 1 to 3 days post-adult emergence (5 to 7 days post-injection). Representative photographs are shown. Metric bar = 1 mm.

**Figure 6:**
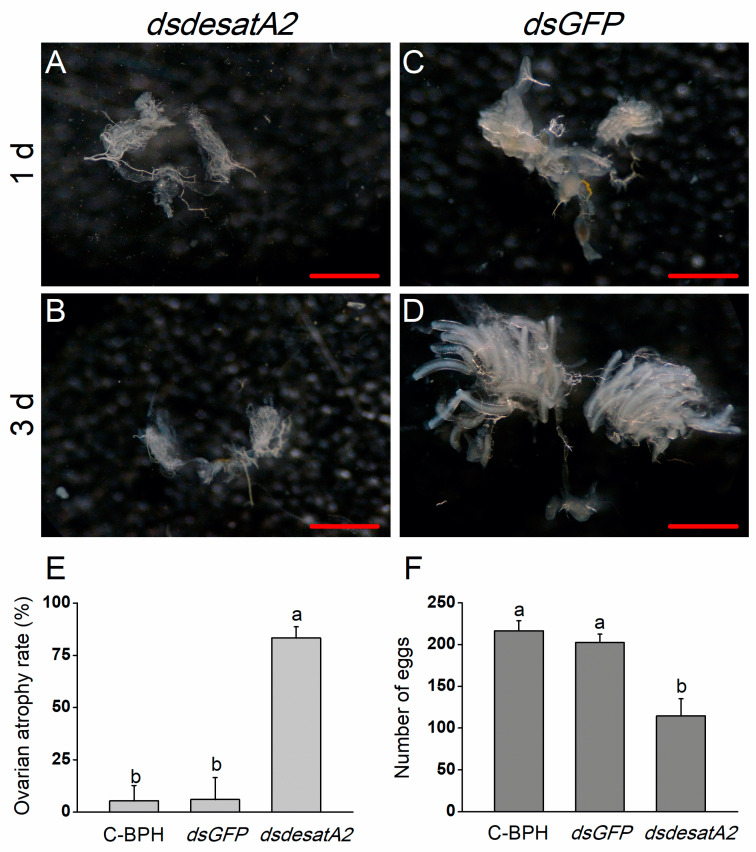
The effects of *Nlug-desatA2*-knockdown on the ovary development and fecundity of female BPH adults. Ovarian phenotypes of 1- (**A**,**C**) and 3-day old (**B**,**D**) female BPH adults which were injected with 0.25 µg dsRNA of *Nug-desatA2* (*dsdesatA2*, (**A**,**B**)) or *GFP* (*dsGFP*, (**C**,**D**)) (third-instar nymphs were injected). Metric bar = 500 µm. (**E**) mean levels (+ SE, *n* = 3) of ovarian atrophy rate among 3-day-old female adults that were injected with 0.25 µg dsRNA of *Nlug-desatA2* (*dsesatA2*) or *GFP* (*dsGFP*), or not injected (C-BPH, control), at the third-instar nymph stage. (**F**) Mean number of eggs (+ SE, *n* = 21) laid on rice plants by a female adult that received the same treatment as in (**E**) at the fifth-instar nymph stage. Letters indicate significant differences among different treatments (*p* ˂ 0.05, Duncan’s multiple range test).

*Nlug-desatA2*-knockdown female adults displayed relatively reduced body size and abdomen atrophy in comparison with dsGFP-BPH females from 1 to 3 days post-adult emergence (Figure 5). When we scrutinized the ovary phenotype in *Nlug-desatA2*-knockdown female adults, we observed that the injection of *dsdesatA2* seems to have blocked ovary development in 1- and 3-day-old female BPH adults (Figure 6A–D), and the ovarian atrophy rate in the dsdesatA2-BPH females was significantly higher (83.3%) than that in the dsGFP-BPH (5.28%) or C-BPH (5.94%) females (Figure 6E). Accordingly, the number of eggs laid by dsdesatA2-BPH females for 10 days (114.4 eggs) was lower than the number of eggs laid by control groups (216.4 and 202.4 eggs in C-BPH and dsGFP-BPH, respectively) (Figure 6F).

**Figure 7:**
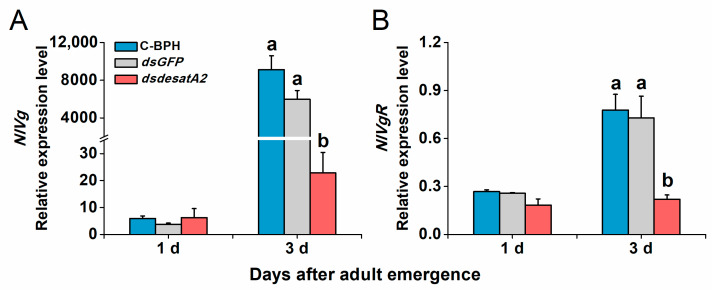
The relative expression level of *NlVg* and *NlVgR* in female BPH adults. Mean transcript levels (+ SE, *n* = 3) of *NlVg* (**A**) and *NlVgR* (**B**) genes of 1- and 3-day-old female adults that were injected with 0.25 µg dsRNA of *Nlug-desatA2* (*dsdesatA2*) or *GFP* (*dsGFP*), or not injected (C-BPH), at the third-instar nymph stage. The results (threshold cycle values) of the qRT-PCR assays were normalized to the expression level of *RPS15* (ribosomal protein S15e, GenBank accession number: ACN79501). Letters indicate significant differences among different treatments (*p* ˂ 0.05, Duncan’s multiple range test).

**Figure 8:**
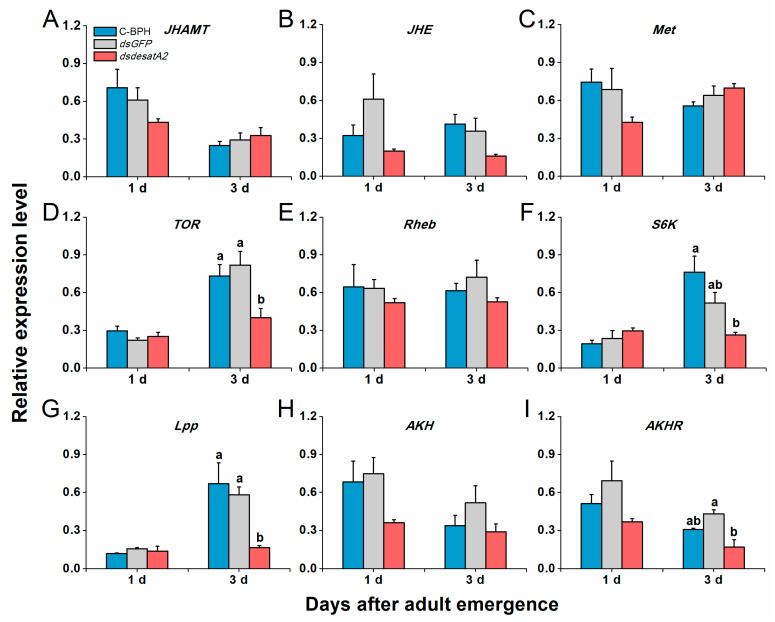
The effects of *Nlug-desatA2*-knockdown on the expression of genes related to JH, TOR and AHK pathways, and lipid transport. Mean transcript levels (+ SE, *n* = 3) of nine genes in 1- and 3-day-old female adults that were injected with 0.25 µg dsRNA of *Nlug-desatA2* (*dsdesatA2*) or *GFP* (*dsGFP*), or not injected (C-BPH), at the third-instar nymph stage. (**A**–**C**) Genes in the juvenile hormone (JH) pathway that regulate JH biosynthesis (juvenile hormone acid methyltransferase, *JHAMT*), JH degradation (juvenile hormone esterase, *JHE*) and receptor of JH (methoprene-tolerant, *Met*). (**D**–**F**) Genes in the signaling pathway that regulate the nutritional target of rapamycin (TOR, *TOR*), the TOR upstream activator (small GTPase Ras homologue enriched in the insect brain, *Rheb*) and the TOR major downstream target (S6 protein kinase, *S6K*). (**G**–**I**) Genes that regulate lipid transport (lipophorin, *Lpp*) and AKH pathway (adipokinetic hormone, *AKH*) and adipokinetic hormone receptor (*AKHR*). Letters indicate significant differences among different treatments (*p* ˂ 0.05, Duncan’s multiple range test).

Transcript levels of *NlVg* and *NlVgR* were elevated in female C-BPH and dsGFP-BPH adults from 1 to 3 days post-adult emergence and suppressed by the knockdown of *Nlug-desatA2* (Figure 7). To examine *Nlug-desatA2*-related fecundity regulation, we evaluated the relative expression levels of eight genes belonging to the JH, TOR and AKH signaling pathways and one primary lipid carrier, the protein gene *Lpp* in dsdesatA2-BPH and control groups (dsGFP-BPH and C-BPH). Transcript levels of *JHAMT* (Figure 8A), *JHE* (Figure 8B) and *Met* (Figure 8C) in the JH pathways were not altered by knocking down *Nlug-desatA2*. Although levels of *TOR* transcripts in 3-day-old females were significantly downregulated (Figure 8D), levels of the upstream activator *Rheb* and the downstream target *S6K* of TOR were not affected by *Nlug-desatA2* knockdown (Figure 8E,F). In lipid transport, an unambiguous increase of *Lpp* transcripts from 1 to 3 days post-adult emergence was hampered by the silencing of *Nlug-desatA2* in 3-day-old-female BPH adults (Figure 8G). On the other hand, lower transcript levels of *AKHR* were found in 3-day-old dsdesatA2 female BPH adults than in that of controls, although no similar difference was observed in *AKH* transcripts (Figure 8H,I).

### 2.4. Herbivory by Nlug-desatA2-Knockdown BPH Increases the Level of SA in Rice

**Figure 9:**
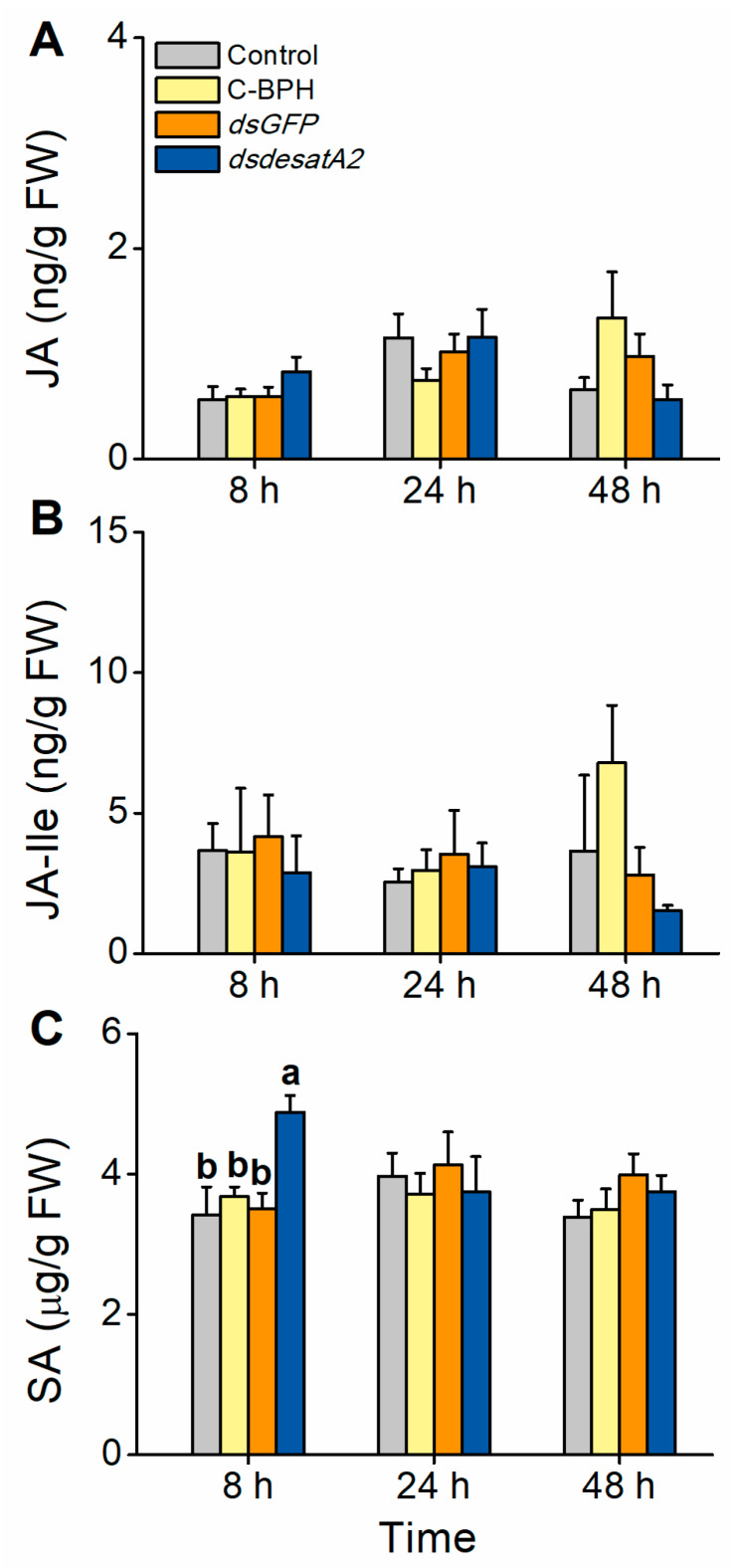
Effect of *Nlug-desatA2*-knockdown BPH feeding on rice plants with levels of jasmonic acid (JA), jasmonoyl-isoleucine (JA-Ile) and salicylic acid (SA). Mean levels (+ SE, *n* = 5) of JA (**A**), JA-Ile (**B**) and SA (**C**) in plants that were not infested (controls) or infested with newly emerged female BPH adults that had been injected with the dsRNA of *Nlug-desatA2* (*dsdesatA2*) or *GFP* (*dsGFP*), or not injected (C-BPH), at the third-instar nymph stage. Due to the difference in food intake between BPH in response to different treatments, 20 (dsdesatA2-BPH) or 10 (dsGFP-BPH and C-BPH) female adults were released onto single seedlings. Letters indicate significant differences among different treatments (*p* ˂ 0.05, Duncan’s multiple range test).

We also evaluated the involvement of *Nlug-desatA2* in the BPH–rice interaction. The JA, JA-Ile and SA signaling pathways play central roles in plant defense responses in many plant species, including rice [56,57,58]. To examine whether feeding by *Nlug-desatA2*-knockdown BPH influenced the defense response in rice plants, we measured the levels of these phytohormones 8, 24 and 48 h post-infestation. The levels of JA and JA-Ile were similar between rice plants infested with 1-day-old female adults in which *Nlug-desatA2* had been knocked down and controls. SA levels at 8 h post infestation were significantly higher in dsdesatA2-BPH-infested plants than in dsGFP-BPH-infested plants, C-BPH plants and control plants (Figure 9).

## 3. Discussion

In this study, we found that the knockdown of *Nlug-desatA2* affected the food intake, survival, development and reproduction of BPH. Moreover, we recorded an increase in the level of SA in dsdesatA2-BPH-infested rice plants at 8 h after infestation, compared to that in control BPH-infested rice plants. These findings demonstrate that *Nlug-desatA2* plays an important role not only in the food intake, survival, development and reproduction of BPH, but also in the BPH–rice interaction.

In *D. melanogaster*, both fatty acid desaturases inhibitor CAY10566-fed and *desat1*-deficient/RNA interference increased the ratio of SFAs versus UFAs and blocked food intake [13,59]. Similarly, here, we found that suppressing *Nlug-desatA2* significantly reduced the concentration of 5 of 7 fatty acids, including 2 SFAs and 3 UFAs, and reduced the ratios of C16:1 to C16:0, C18:1 to C18:0 and MUFAs to SFAs in 3-day-old BPH females (Figure 1 and Figure S3). Moreover, knocking down *Nlug-desatA2* reduced the food intake of BPH (Figure 3A). These data demonstrate that like *desat1* in *D. melanogaster*, *Nlug-desatA2* in BPH functions as a desaturase, introducing a double bond into fatty acyl substrates between the ninth and tenth carbon molecules; in addition, *Nlug-desatA2* plays an important role in regulating the ratio of SFAs to UFAs and food intake in BPH. It has been reported that the decrease in the ratio of UFAs to SFAs in *D. melanogaster* results in larval food avoidance by influencing the insect’s lipid-sensor system [13]. Hence, the reduction in food intake in *Nlug-desatA2*-knockdown BPH, compared to controls, is probably related to the decrease in the ratio of UFAs to SFAs. Interestingly, we also observed that the concentration of 2 SFAs and 3 UFAs as well as of total fatty acids and TAGs in *Nlug-desatA2*-knockdown BPH decreased. This decrease may have resulted from the low ratio of MUFAs to SFAs. The low ratio likely caused the decrease in food intake of BPH as stated above, and low food intake may have switched on a metabolic compensation mechanism, causing stored energy substances, including fatty acids and TAGs, to be consumed, and also directly decreased the level of TAGs, as was found in *A. coluzzii* with *SCD1* knockdown [15]. Future research should elucidate these issues.

In addition to influencing the food intake of BPH, *Nlug-desatA2* knockdown also reduced its survival, growth and egg production, and caused both severe abdomen atrophy (Figure 5) and ovarian agenesis (Figure 6). Consistently, we also observed a drastic reduction in the transcript levels of *NlVg* (Figure 7A) and *NlVgR* (Figure 7B) after *Nlug-desatA2*-knockdown. Similar phenomena have also been reported in *D. melanogaster* [13,59] and *A. coluzzii* [15]. In the latter, for example, the knockdown of *SCD1* enhanced the mortality of larvae and inhibited egg development in female adults [15]. Overloading a cell with SFAs can induce a toxic response known as ‘‘lipotoxicity,’’ which causes a variety of diseases and dysfunctions in humans and animals, including insects [15,60]. The midgut cell membranes of *SCD1*-knocked down *A. coluzzii*, for example, are thick and rigid, and failed to extend after a blood meal. These midgut cells contained indistinct and irregularly shaped mitochondria, with few lipid droplets [15]. Moreover, TAG is the principal form of stored lipids in insects, and the lipid stores in insect oocytes are the main source of energy for developing embryos [15]. Hence, the lipotoxicity and decreased food intake of BPH caused by the lower ratio of UFAs to SFAs, and the accompanying indirect effects, such as the decrease in fatty acids and TAG levels in BPH, may explain why the knockdown of *Nlug-desatA2* decreased survival, growth and fecundity in BPH, and caused abdomen atrophy and ovarian agenesis.

In *A. coluzzii*, the knockdown of *SCD1* reportedly influences transcript levels of some genes related to pathways regulating JH, nutrition-related TOR, AKH and lipid transport. These pathways may be involved in how SCD1 regulates the fecundity of *A. coluzzii* [15]. We found that the knockdown of *Nlug-desatA2* significantly reduced the mRNA level of *TOR*, *Lpp* and *AKHR* (Figure 8D,G,I), whereas the transcript levels of three genes involved in the JH signaling pathway in BPH (Figure 8A–C) were unaffected. These data suggest that the TOR and AKH pathways, as well as the lipid transport, are involved in the reduction of BPH fecundity after *Nlug-desatA2*-knockdown and that *Nlug-desatA2* knockdown did not influence the transcript level of JH pathway-related genes, *JHAMT*, *JHE* and *Met* in BPH. Whether *Nlug-desatA2* influences the JH pathway by mediating other components and thereby regulates BPH fecundity needs to be elucidated in the future.

Interestingly, we observed that the corrected survival rate of *Nlug-desatA2*-knockdown BPH fed an artificial diet was higher than that of dsdesatA2-BPH fed on rice (Figure 4C). This result suggests the potential involvement of *Nlug-desatA2* in the BPH–rice interaction. Indeed, we recorded higher SA levels in rice seedlings infested by dsdesatA2-BPH than in those infested by dsGFP-BPH or controls at 8 h after BPH infestation (Figure 9C). The SA signaling pathway has been reported to confer resistance to BPH on rice [61]. Thus, the high corrected mortality observed in dsdesatA2-BPH fed on rice is probably related to the accumulation of SA in rice. Why did infestation of dsdesatA2-BPH induce higher levels of SA in rice than did infestation of dsGFP-BPH or control BPH at 8 h? Although the number of dsdesatA2-BPH on each plant, in the SA measurement experiment, was 2-fold higher than the number of control BPH, the damage of individual plants caused by dsdesatA2-BPH or C-BPH was similar as the food intake of dsdesatA2-BPH individuals was about half of the food intake of C-BPH individuals. Thus, the difference in SA levels between dsdesatA2-BPH-infested plants and C-BPH-infested plants should have resulted from the changes in physiological and biochemical status of BPH caused by the knockdown of *Nlug-desatA2*, not from the difference in damage levels. These changes may affect the level or activity of BPH salivary elicitors and/or effectors, thereby influencing the defense response in rice. In BPH, several salivary elicitors and effectors, such as NlMLP (*N. lugens*-secreted mucin-like protein) [62], NlEG1 (*N. lugens* salivary endo-β-1,4-glucanase) [63] and NlSEF1 (*N. lugens* salivary EF-hand calcium-binding protein) [47], have been reported. Further characterization will be necessary to determine the role of *Nlug-desatA2* in the BPH–rice interaction.

Interestingly, Li et al. [64] reported that BPH infestation could induce a weak but significant increase in SA levels in TN1 plants 24 and 48 h post infestation. However, in this study, we did not find an obvious change in SA levels in TN1 plants infested with C-BPH (Figure 9C). This discrepancy might be because of different growth conditions and developmental stages of plants as well as different populations of BPH used in the two studies. These factors have been well documented to influence defense-related signaling pathways and their downstream defense responses in plants [40,42,65].

In conclusion, our data show that *Nlug-desatA2* catalyzes the formation of unsaturated bonds between the ninth and tenth carbon molecules in fatty acyl substrates in BPH. It plays an important role in the food intake, growth, survival and reproduction of BPH by regulating the ratio of MUFAs to SFAs. Importantly, *Nlug-desatA2* may also be indirectly involved in regulating the defensive responses of rice to infestation by BPH. These results demonstrated that *Nlug-desatA2* can serve as a potentially useful target for RNAi-based pest management.

## 4. Materials and Methods

### 4.1. Plant Growth and Insect Rearing

The rice variety Taichun Native 1 (TN1) was used in these experiments. Plants were grown as described by Lu et al. [57]. Thirty-day-old seedlings were individually planted in 500 mL hydroponic plastic pots with rice nutrient solution [66]. The brown planthopper (BPH), *Nilaparvata lugens*, was initially provided by the Chinese National Rice Research Institute (Hangzhou, China) and reared on fresh TN1 seedlings in a phytotron (27 ± 1 °C and 70% ± 10% relative humidity under a 14/10 h light/dark photoperiod) in Zhejiang University (Hangzhou, China).

### 4.2. RNAi Experiment

A 287 bp fragment of *Nlug-desatA2* (GenBank accession number: MH271234) and an 860 bp fragment of control gene *GFP* were amplified by RT-PCR with primers, including a T7 promoter sequence (Table S1). The dsRNAs were synthesized with PCR products by using a MEGAscript T7 High-Yield Transcription Kit (Ambion, Austin, TX, USA). Third- or fifth-instar nymphs were injected as described in Reference [55], with 0.25 µg dsRNA of *Nlug-desatA2* or *GFP*, and controls were not injected (C-BPH). The silencing efficiency of *Nlug-desatA2* in the whole bodies of BPH adults was investigated on 1 and 3 days post-adult emergence (5 and 7 days post-injection if nymphs were injected at the third instar, or 2 and 4 days post-injection if nymphs were injected at the fifth instar. RNA was extracted from 15 female individuals).

### 4.3. Real-Time qPCR Analysis

Total RNA was extracted from whole bodies of 1- and 3-day-old BPH female adults by using the SV Total RNA Isolation System (Promega, Madison, WI, USA) following the manufacturer’s instructions. The cDNA was prepared with 500 ng of each RNA sample by using a Takara PrimeScript™ RT reagent kit. To investigate the possible function of *Nlug-desatA2* during ovary development in BPH female adults, qRT-PCR was performed using specific primers of eleven genes related to fecundity (Table S2) on the CFX96™ Real-Time system (Bio-rad, Hercules, CA, USA) with iQ SYBRGreen Supermix (Bio-Rad). Relative expression levels of each gene were normalized with the *RPS15* gene (ribosomal protein S15e, GenBank accession number: ACN79501) [67] and calculated using the 2^−^^ΔΔCt^ method [68]. Three independent biological replicates were analyzed.

### 4.4. Fatty Acid Analysis

Total lipids were extracted from groups of 1- and 3-day-old BPH female adults (n = 15) at 5 or 7 days post-injection of dsRNA of *Nlug-desatA2* or *GFP* (third-instar nymphs were injected). Samples were homogenized in 600 µL petroleum ether and then centrifuged at 10,000× *g* for 5 min at 4 °C. The supernatant was transferred into pre-weighed centrifuge tubes, evaporated at 30 °C for 5 min to dryness by a rotavapor and re-weighed. The crude fat was mixed with 600 µL reaction reagent (200 µL hexane, 200 µL methanol and 200 µL KOH-methanol (0.8 mol/L)), inverted for 5 min, and then vibrated for another 5 min after adding 200 µL ultra-pure water. One hundred microliters of the supernatant were collected. We added 10 µL methyl heptadecanoate (4 µg/µL) as the internal standard to 100 µL of the supernatant and measured the concentration of fatty acid methyl esters (FAMEs) in each sample to represent fatty acid concentrations. The FAMEs were analyzed on an Agilent GC6890 gas chromatography equipped with Agilent DB-23 column (60 m × 0.25 mm, internal diameter 0.15 µm) and identified by using Supelco^®^ 37 Component FAME Mix. Chromatography conditions were described in Reference [54]: 250 °C inlet temperature, 242.3 kPa, splitless, 1 µL injection volume, N_2_ carrier gas (30 mL/min), H_2_ flow 40 mL/min, airflow 445 mL/min. Oven temperature was held at 50 °C for 1 min, increased at 25 °C/min to 175 °C, then at 3.5 °C/min to 230, and finally, held at 230 °C for 5 min. Three biological replicates were analyzed.

### 4.5. Triglyceride Quantification

For triglyceride (TAG) measurement, 1- and 3-day-old BPH female adults (5 and 7 days post-injection, third-instar nymphs were injected, *n* = 3) were weighed and then homogenized in 200 µL Tris-EDTA-Triton X-100 (TET) buffer (10 mM Tris-HCl, pH 8.0, 1 mM ethylenediaminetetraacetic acid (EDTA), 0.1% Triton X-100), placed in a water bath at 70 °C for 10 min and centrifuged at 10,000 rpm for 10 min at 4 °C. TAGs were determined by using the Free Glycerol Detection Kit (Sigma-Aldrich, St. Louis, MO, USA) in combination with Triglyceride Reagent (Sigma -Aldrich, St. Louis, MO, USA) following the manufacturer’s instructions. Levels of TAGs were calculated based on three replicates.

### 4.6. BPH Bioassays

To examine the phenotypes of *Nlug-desatA2*-knockdown BPH, 1- to 3-day-old female adults injected with the dsRNA of *Nlug-desatA2* (dsdesatA2-BPH) or *GFP* (dsGFP-BPH) (third-instar nymphs were injected) were collected and examined under a microscope. For each treatment, the phenotypes of more than 60 females were observed. To investigate ovarian atrophy in 3-day-old female adults, the ovaries of dsdesatA2-BPH, dsGFP-BPH and C-BPH were dissected (a total of more than 60 female individuals for each treatment), and the ovarian atrophy rate was analyzed based on three replicates (Table S3). The effect of *Nlug-desatA2*-knockdown on BPH fecundity was also investigated. Stems of intact rice plants (one plant per pot) were individually confined within glass cylinders (diameter 4 cm, height 8 cm, with 48 small holes, diameter 0.8 mm) into which one newly emerged dsdesatA2-BPH, dsGFP-BPH or C-BPH female adult (fifth-instar nymphs were injected) and one untreated newly emerged BPH male adult were released. The number of eggs laid by females on each rice plant 10 days after the release was counted under the microscope. A new male adult was released on the seedling if the original male had died during the experiment. At the end of the experiment, the plant was discarded if the female had not survived. The experiment was repeated 21 times.

To test whether *Nlug-desatA2*-knockdown influences BPH food intake, a newly emerged dsdesatA2- or dsGFP- (third-instar nymphs were injected) or C-BPH female adult was released into a parafilm bag (6 × 5 cm) fixed on the stem of a single rice plant. The honeydew excreted by BPH as an index of food intake [69] was weighed 24 h after the insects were released. The experiment was repeated 19 times. To compare body weight, five 1- to 3-day-old female adults injected with dsRNA of *Nlug-desatA2* or *GFP* (third-instar nymphs were injected), or non-injected controls, were collected and weighed. Each treatment was replicated six times.

To measure survival rates of BPH, third-instar nymphs injected with *Nlug-desatA2* or *GFP* dsRNA (controls were not injected) were allowed to feed on rice plants or artificial diet. Stems of rice seedlings (one plant per pot) were individually confined within glass cylinders, as stated above, into which 20 third-instar nymphs were released. For the artificial diet test, 20 third-instar nymphs were released into individual feeding chambers as described by Fu et al. [70]. The number of nymphs that survived was recorded every day for ten days. Each treatment had five replicates. The survival rate of BPH with different treatments (dsdesatA2-BPH, dsGFP-BPH or C-BPH) on rice plants or artificial diet was calculated. Moreover, to adjust for control mortality, the corrected survival rate of dsdesatA2-BPH, using the *dsGFP*-injected group as the control, fed on rice variety TN1 or an artificial diet was also calculated using the same method as described in Zhao et al. [71].

### 4.7. JA, JA-Ile and SA Analysis

Rice plants were infested by BPH that had been injected with dsRNA of *Nlug-desatA2* (dsdesatA2-BPH) or *GFP* (dsGFP-BPH) (third-instar nymphs were injected), or by non-injected BPH (C-BPH). The stems of rice plants were individually confined in glass cylinders, into which 20 (dsdesatA2-BPH) or 10 (dsGFP-BPH and C-BPH) newly emerged female adults had been released. The number of BPH with different treatments, dsdesatA2-BPH, dsGFP-BPH or C-BPH, on each plant was determined according to the difference in their food intakes on plants (Figure 3A; ensuring that each plant received similar damage from BPH). Outer sheaths of the stem were harvested at 8, 24, and 48 h post-infestation. Non-infested plants were used as controls. JA, JA-Ile and SA levels in the sheath were determined individually by using high-performance liquid chromatography-mass spectroscopy with labeled internal standards (^2^D_6_-JA, ^2^D_6_-JA-Ile and ^2^D_4_-SA) following the method of Lu et al. [72]. Each treatment was replicated five times.

### 4.8. Data Analysis

Statistical analyses were carried out by using the PASW^®^ Statistics 18 and Prism 8.0.2 software. Differences between two treatment groups were analyzed by Student’s *t*-test. Differences in experiments involving three treatments were analyzed by one-way analysis of variance (ANOVA). Duncan’s multiple range tests were followed if the ANOVA was significant (*p* < 0.05). Differences in BPH survival rates between treatments were analyzed by using the log-rank (Mantel–Cox) test.

## Supplementary Materials

Supplementary materials can be found at https://www.mdpi.com/1422-0067/21/11/4143/s1. Figure S1: The silencing efficiency of *Nlug-desatA2* by RNAi. Figure S2: The effect of *Nlug-desatA2*-knockdown on the level of total saturated and unsaturated fatty acids. Figure S3: The effect of knocking down *Nlug-desatA2* on the desaturase indices in BPH. Table S1: Primers used for dsRNA synthesis. Table S2: Primers used for qRT-PCR. Table S3: Knockdown of *Nlug-desatA2* leads to ovarian atrophy phenotype of female BPH adults.
