# Supplementary material for: The Desaturase Gene Nlug-desatA2 Regulates the Performance of the Brown Planthopper Nilaparvata lugens and Its Relationship with Rice
Source: Int J Mol Sci. 2020 Jun 10;21(11):4143. doi: 10.3390/ijms21114143 (PMC7312190; doi:10.3390/ijms21114143)
Supplement: Supplementary file 1 [file ijms-21-04143-s001.pdf]

International Journal of
*Molecular Sciences*

MDPI

*Supplementary Materials*

# The Desaturase Gene *Nlug-desatA2* Regulates the Performance of the Brown Planthopper *Nilaparvata lugens* and Its Relationship with Rice

**Wen-feng Ye, Jia-mei Zeng, Wen-hui Hu, Carlos Bustos-Segura, Ali Noman, Yong-gen Lou***

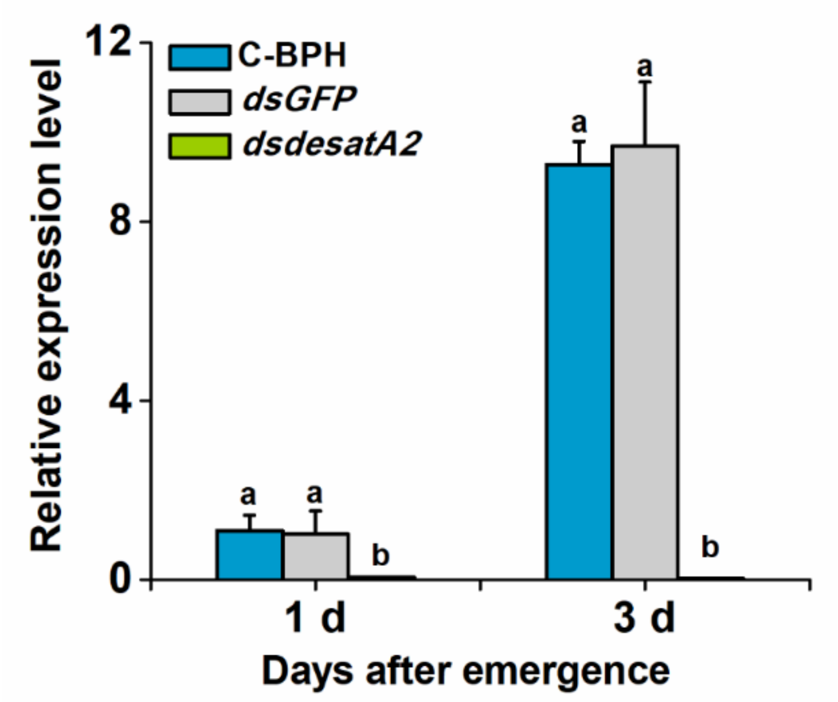


**Figure S1.** The silencing efficiency of *Nlug-desatA2* by RNAi. Mean transcript levels (+ SE, n = 3) of *Nlug-desatA2* in whole bodies of newly emerged and 3-day-old BPH female adults that were injected with dsRNA of *Nlug-desatA2* or *GFP* (*dsGFP*), or not injected (C-BPH) at third nymph instar (5 and 7 days after injection). The results (threshold cycle values) of the qRT-PCR assays were normalized to the expression of *RPS15*. Letters indicate significant differences among different treatments ($P < 0.05$, Duncan's test).

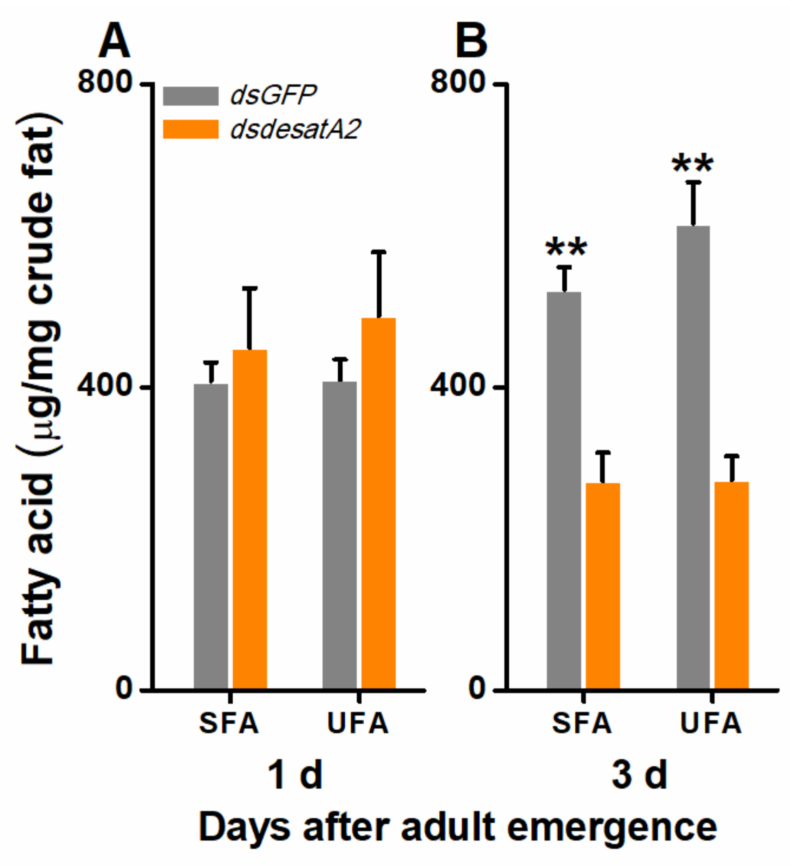


**Figure S2.** The effect of *Nlug-desatA2*-knockdown on the level of total saturated and unsaturated fatty acids. Mean levels (+ SE, n = 3) of fatty acids per milligram crude fat from the whole body of 1- (A) and 3-day-old (B) female adult that were injected with 0.25 μg dsRNA of *Nlug-desatA2* (*dsdesatA2*) or *GFP* (*dsGFP*) at the third-instar nymph stage. SFA, saturated fatty acids; UFA, unsaturated fatty acids. Asterisks indicate significant difference between *dsGFP* and *dsdesatA2* injection. (** $P < 0.01$, Student's t test).

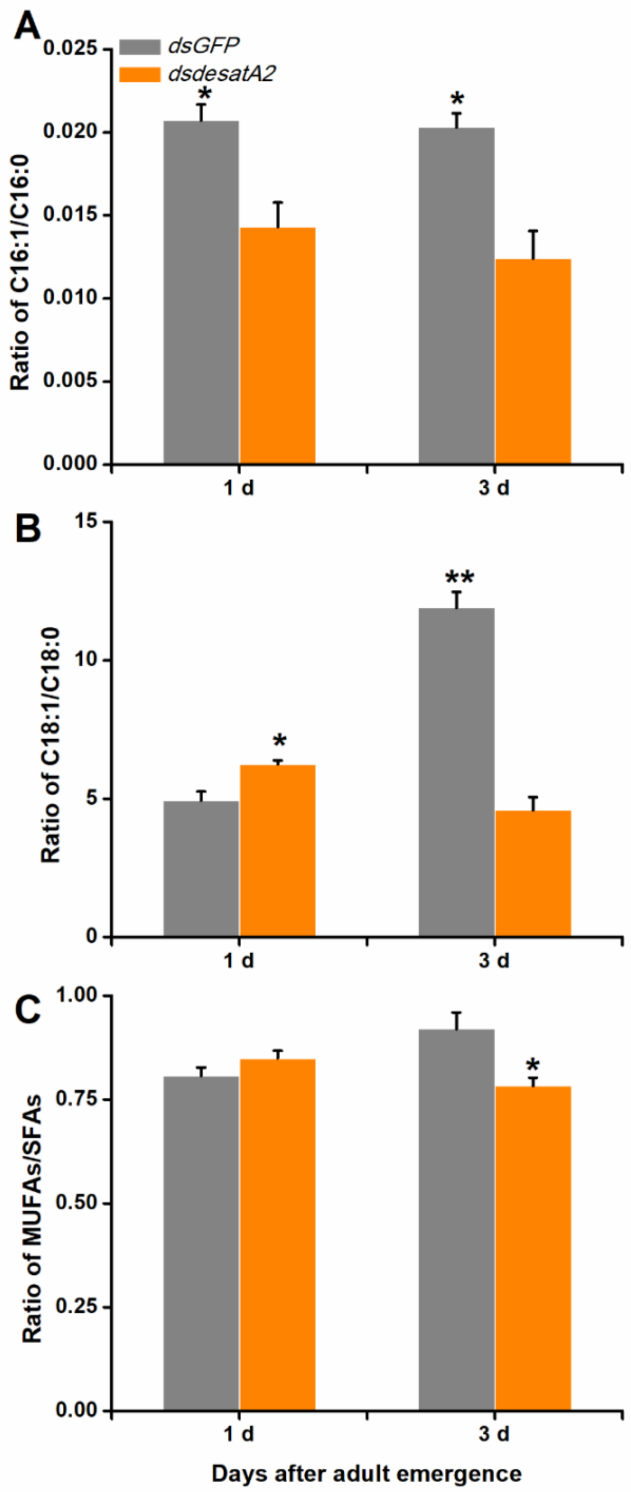


**Figure S3.** The effect of knocking down *Nlug-desatA2* on the desaturase indices in BPH. The ratio of C16:1 to C16:0 (A), C18:1 to C18:0 (B) and MUFAs to SFAs (C) in the whole bodies of 1- and 3-day-old female adults that had been injected with 0.25 μg dsRNA of *Nlug-desatA2* (*dsdesatA2*) or *GFP* (*dsGFP*) at the third-instar nymph stage. Asterisks indicate significant difference between treatments (* $P < 0.05$, ** $P < 0.01$, Student's $t$-test).

**Table S1.** Primers used for dsRNA synthesis.

| Primers | Primer Sequences (5'-…-3') |
|---|---|
| *Nlug-desatA2*-F | GGATCCTAATACGACTCACTATAGGGTGGAGAAATGTTGCTGCCT |
| *Nlug-desatA2*-R | GGATCCTAATACGACTCACTATAGGCGGTGTCTGTGAACTTGTGG |
| *GFP*-F | GGATCCTAATACGACTCACTATAGGAAGGGCGAGGAGCTGTTCACCG |
| *GFP*-R | GGATCCTAATACGACTCACTATAGGCAGCAGGACCATGTGATCGCGC |

F: forward primer; R: Reverse primer.

**Table S2.** Primers used for qRT-PCR.

| Gene | Genbank accession number | Forward primer (5'-…-3') | Reverse primer (5'-…-3') |
|---|---|---|---|
| *Nlug-desatA2* | MH271234 | CTGGAGCAGAAGTCATCCCT | CGCTGTTGTTGTTGTTGTTG |
| *RPS15* | ACN79501 | TAAAAATGGCAGACGAAGAGCCCAA | TTCCACGGTTGAAACGTCTGCG |
| *NlVg* | AB353856 | ATGAGTTCAACCCAGCATCA | GATGAGCTTGAGCTGCTGTC |
| *NlVgR* | GU723297 | AGGCAGCCACACAGATAACCGC | AGCCGCTCGCTCCAGAACATT |
| *JHAMT* | KP769805 | GAACCTGCAGGCCAAACACA | ACCACTCGGTTGGGCTGAAT |
| *JHE* | EU380769 | AAGTAACTGGCAGATTCAACC | CTCGAATAGATGTGCTGCAGG |
| *Met* | KP797880 | GGTGGTAAACGGATTGGAAA | CATCGTCAGCCAACTCGATA |
| *Tor* | JQ793898 | GGCTACAGGGATGTCAAA | GAGATAGATTCAAACGGAAAG |
| *Rheb* | JX175249 | ACGGCGGGTCAGGATGAGTA | TCGAAGGACTTGGATGAGGTGA |
| *S6K* | KP769804 | AGGACACTGCGCACACCAAG | CAATTCTCCGCCGCTCAGAT |
| *Lpp* | AB465596 | GACGGTCACTATGTGTTGGC | CAACACTGTCCACCCTATCG |
| *AKH* | JQ082123 | AGCTGATAAGATGGCTCGTGCT | TGGATGCCTTGCAGCCTTCT |
| *AKHR* | MH238458 | TCGCATGCACCAACTCTTGC | CGCTGCGTTTGAGAGCCATT |

**Table S3.** Knockdown of *Nlug-desatA2* leads to ovarian atrophy phenotype of female BPH adults.

| Treatment | Repeat* | Ovarian Atrophy (n) | Normal Ovary (n) | All (n) | Ratio (Ovarian Atrophy/All) (%) | Mean Ratio (%) | SE |
|---|---|---|---|---|---|---|---|
| | 1 | 1 | 23 | 24 | 4.17 | | |
| C-BPH | 2 | 1 | 19 | 20 | 5.00 | 5.28 | 0.73 |
| | 3 | 2 | 28 | 30 | 6.67 | | |
| | 1 | 1 | 21 | 22 | 4.55 | | |
| *dsGFP* | 2 | 1 | 18 | 19 | 5.26 | 5.94 | 1.05 |
| | 3 | 2 | 23 | 25 | 8.00 | | |
| | 1 | 16 | 3 | 19 | 84.21 | | |
| *dsdesatA2* | 2 | 28 | 6 | 34 | 82.35 | 83.30 | 0.54 |
| | 3 | 20 | 4 | 24 | 83.33 | | |

* Three experiments.
